# Evaluation of intraoperative subcutaneous anesthetic infiltration for pain management in patients undergoing total thyroidectomy^☆^^☆☆^

**DOI:** 10.1016/j.bjorl.2025.101613

**Published:** 2025-05-27

**Authors:** Phamella Carlesse, Nathalia Cardoso, Tamara Harati, Bruna Lapichini, Daniel Herman Partezani, Leandro Luongo de Matos, Rogério Aparecido Dedivitis

**Affiliations:** a Universidade Metropolitana de Santos, Santos, SP, Brazil; b Irmandade Santa Casa de Santos, Departamento de Cirurgia de Cabeça e Pescoço, Santos, SP, Brazil; c Faculdade de Medicina da Universidade de São Paulo (FMUSP), Departamento de Cirurgia de Cabeça e Pescoço, São Paulo, SP, Brazil

**Keywords:** Pain, Thyroid diseases, Thyroidectomy, Bbupivacaine, Anesthetic infiltration

## Abstract

**Objective:**

To evaluate the efficacy of intraoperative Bupivacaine application in reducing postoperative pain among patients undergoing total thyroidectomy.

**Methods:**

A prospective study was carried out with 153 female patients undergoing total thyroidectomy, with a randomization protocol for topical anesthetic infiltration. Postoperative pain intensity was self-reported by the patients using the Visual Analog Scale (VAS) at the 1st, 6th, and 24th postoperative hours.

**Results:**

Comparative analysis revealed that patients who did not receive anesthetic infiltration experienced, on average, higher pain levels during all three observation periods (1st and 6th hours, and 24th hour; p = 0.0001 – repeated measures ANOVA multiple test) relative to those who received the anesthetic. Isolated observations showed that pain was significantly less severe at the 24th hour compared to the 6th hour (p = 0.001 – repeated measures ANOVA test).

**Conclusion:**

Topical anesthetic infiltration proves to be an effective tool for managing post-thyroidectomy pain.

**Level of evidence:**

Level III.

## Highlights

• Patients who received anesthetic infiltration had lower pain levels;
• The pain dropped along the first postoperative day;
• Local anesthetics can reduce the necessity of opioids postoperatively.

## Introduction

Postoperative pain in thyroid surgery is typically of moderate intensity, and up to 90% of patients need analgesics on the first day after the operation.^1^ The perception of postoperative pain likely includes many components connected to the deep and superficial layers of the surgical wound, neck position, and drainage. The average pain level, using a Visual Analog Scale (VAS) varying from 0 to 10, was reported to be 6.9.^1^, ^2^

Wound incisions, hyperextension of the neck, retraction of the wound edges during the operation, and dissection are the main causes of pain. Strategies to minimize pain in the postoperative period, and thereby minimize the use of narcotics, have been explored. Techniques such as multimodal analgesia, local anesthetic infiltration, and superficial or deep cervical block have been employed to relieve pain in the postoperative period of thyroid surgery.^3^, ^4^, ^5^

The combination of 0.75% Ropivacaine and 8 mg of Lornoxicam has been found to improve postoperative pain control and patient comfort while reducing the need for opioids. Bupivacaine, as a long-acting local anesthetic, has been used successfully for postoperative local infiltration. Ropivacaine, a long-acting amide-type local anesthetic, is chemically related to Bupivacaine; however, its toxicity induces cutaneous vasoconstriction, which restricts the systemic absorption of the drug and enlarges the area of anti-inflammatory activity. It may further reduce pain when administered locally.^6^

Although pain after thyroidectomy is not considered very intense and can be easily controlled, a certain degree of dysphagia is usually noticed. Thus, the administration of anesthetic drugs through a non-oral route that does not demand swallowing is recommended. Recent studies have shown that postoperative pain can be reduced by preventive infiltration at the incision site with a local anesthetic.^7^, ^8^ In contrast, a randomized study reported that this same administration of local anesthetic does not influence pain reduction in post-thyroidectomy patients undergoing general anesthesia.^9^

Given these divergences related to the role of local anesthesia in pain management after thyroid surgery, the objective is to evaluate the impact of intraoperative subcutaneous administration of bupivacaine on postoperative pain in patients undergoing total thyroidectomy.

## Methods

This study was approved by the Institutional Review Board (IRB) under the reference number 730.549, dated June 30th, 2014.

A prospective study was carried out with female patients who underwent total thyroidectomy at the Head and Neck Service of the Irmandade da Santa Casa da Misericórdia de Santos. Patients were consecutively enrolled from July to December 2014. Topical anesthetic infiltration was administered every other surgical day, alternating with days when no anesthetic was infiltrated. Thus, patients were divided into two groups: Group A – patients who had received the local anesthetic Bupivacaine 5 mg with Epinephrine 1:200,000, which were aspirated in a 10 mL syringe using a 27 G 3½ needle and infiltrated into the subcutaneous cellular tissue immediately after the placement of the surgical drapes; Group B – patients who did not receive any infiltration. The same standard of analgesia was performed by The anesthesia team applied a consistent analgesia standard in pre-extubation: Dipyrone 20 mg/Kg and Parecoxib 40 mg. No additional superficial cervical plexus blocks were performed.

Parameters compared and analyzed between the two groups included age, size of the goiter (volume in milliliters measured by preoperative ultrasound), incision size (in centimeters), histopathological report (categorized as benign, papillary carcinoma, or other malignant tumors), surgical time (in minutes), postoperative complications (e.g., infection, hematoma, transient or permanent vocal fold paralysis, and transient and persistent hypoparathyroidism), and hospital discharge time (in days). The inclusion criterion was female patients.

**Fig. 1:**
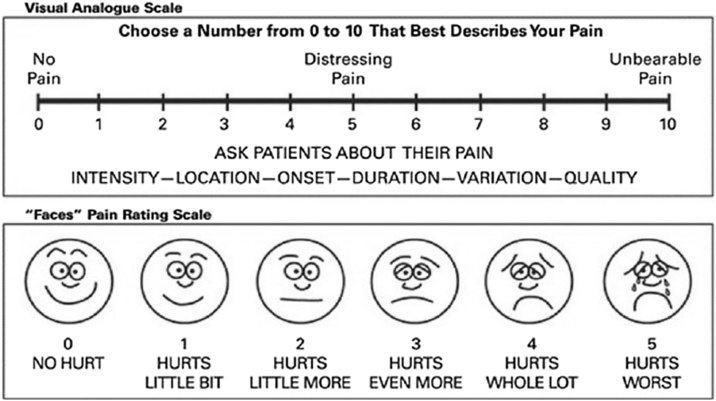
Visual Analogue Scale (VAS) for pain assessment.

The VAS was used to assess pain levels at three periods: 1 -h post-surgery, 6 -hs post-surgery, and at medical discharge, which was 24 -hs after surgery (Fig. 1). All participants received postoperative analgesia in the form of intravenous dipyrone every 6 -hs.

Quantitative data from parametrically distributed variables were organized and described as mean and standard deviation. Qualitative variables were expressed in absolute and relative frequencies. Distributions were defined as parametric according to the Kolmogorov-Smirnov test. The Student’s *t*-test was employed to compared means between two independent samples, whereas the repeated measures ANOVA was used to compare dependent means. For frequency comparisons between groups of qualitative variables, the Chi-Squared test was applied. Statistical analyses were conducted using the SPSS 26.0 software (SPSS® Inc; Illinois, USA). A statistical significance level of less than 5% (p < 0.05) was adopted for all comparisons.

## Results

A total of 153 female patients who underwent total thyroidectomy were analyzed. The average age was 41.8 years (18–77 range). The average volume of the goiter was 33.9 cm^3^, and the average incision size was 5 cm. Homogeneity tests were carried out, confirming that the casuistries were homogeneous between the two groups regarding age, goiter volume, incision size, and other evaluated aspects (Table 1).

**Table 1:** Series distribution and homogeneity analysis.

Factor	Aspect	Group A (n = 98)	Group B (n = 55)	p-value
Age (years)	Mean	41.14	42.93	0.443
	Standard deviation	14.269	13.326	
	Median	38	42	
	Minimum	18	22	
	Maximum	77	75	
Goiter volume (mL)	Mean	36.142	38.356	0.79
	Standard deviation	4.9225	6.6861	
	Median	17.9	18.0	
	Minimum	4.0	6.5	
	Maximum	246.3	245.3	
Incision size (cm)	Mean	4.913	5.16	0.259
	Standard deviation	0.1156	0.1839	
	Median	5.0	5.0	
	Minimum	3.0	3.5	
	Maximum	8.0	9.0	
Histopathological report	Benign	72	41	N/S
	Papillary carcinoma	24	13	
	Other malignancies	2	1	
Surgical time (minutes)	Mean	86	84	N/S
Postoperative complications	Infection	0	0	N/S
	Hematoma	0	0	
	Transient vocal fold paralysis	2	1	
	Permanent vocal fold paralysis	0	0	
	Transient hypoparathyroidism	3	1	
	Persistent hypoparathyroidism	1	1	
	Infection	0	0	
Discharge time (days)	Mean	1.05	1.1	N/S

Group A: With Bupivacaine injection; Group B: Without Bupivacaine injection; Student test; N/S, No statistical significance noted.

To compare the pain intensity among patients, anesthetic infiltration (Bupivacaine) was performed in 98 women (Group A), while 55 patients did not receive the infiltration (Group B). The VAS was applied to classify pain and showed an average pain intensity of 1.48 for patients who underwent infiltration and 5.05 for those who did not at the 1st postoperative hour (p < 0.001 – Student’s *t*-test). At the 6th postoperative hour, the average pain intensity was 1.63 for the patients who received infiltration and 3.49 for those who did not (p < 0.001 – Student’s *t*-test). Finally, upon discharge after 24 h, patients who underwent infiltration reported an average pain intensity of 1.11, whereas those who did not reported an average pain intensity of 1.96 (p = 0.001 – Student’s *t*-test).

**Fig. 2:**
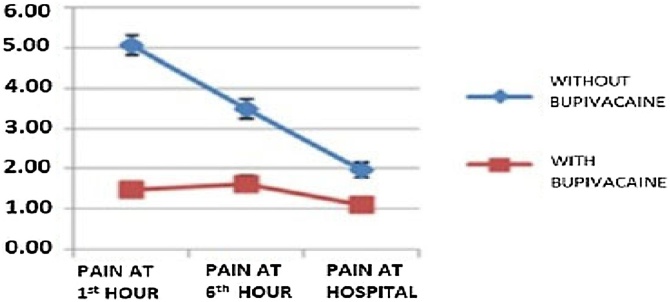
Comparative pain intensity across all observation periods. Throughout all periods observed, the average pain intensity remained higher in the group that did not receive anesthetic infiltration with Bupivacaine.

Analyzing groups separately, in the group that did not receive the anesthetic infiltration, pain gradually subsided throughout the three observation periods (p < 0.0001 – repeated measures ANOVA); in the group that received the intervention, pain levels remained low and stable between the 1st and the 6th hour (p = 1.000 – repeated measures ANOVA) and dropped significantly in the 24th hour compared to the two former periods (p = 0.001 – repeated measures ANOVA). Throughout all observation periods, the average pain intensity was consistently higher in the group that did not receive anesthetic infiltration with Bupivacaine (p < 0.0001 – repeated measures ANOVA) (Fig. 2).

## Discussion

Our study showed that patients who received local anesthetic infiltration with Bupivacaine experienced lower average pain intensity in the early postoperative period than their counterparts who did not receive it.

Pain management after thyroid surgery not only improves patients’ quality of life but also allows a quick return to normal daily activities. Most patients need opioids, which are associated with multiple side effects, such as respiratory depression, sedation, nausea, and vomiting. Consequently, the use of non-opioid agents has been recommended.^8^ The application of local anesthetics has become an important aspect of pain management in surgical environments and is currently recommended in pain management guidelines, reducing the use of opioids in the postoperative period.^10^ Wound infiltration with local anesthetic is an alternative and acceptable method to control postoperative pain. Its main limitation is that only long-acting topical anesthetics deliver effective and sufficiently prolonged analgesia. The local injection of Bupivacaine, which blocks the superficial branches of the cervical plexus, has been successfully employed in local infiltration after surgery, thus forming our rationale for its use in our intervention group.

In a study involving 40 consecutive patients who were randomly assigned to two homogeneous groups: Group I (n = 20) consisted of patients who received Bupivacaine 0.5% (10 mL) wound infiltration at surgery completion, while Group II (n = 20) included patients without infiltration. Postoperative pain medication included Morphine, administered either IV or IM, as needed. Twenty-four hours after surgery, the highest pain was recorded using a visual analogue scoring system, ranging from 0 to 10. The pain scores were significantly different between the two groups. In Group I, the mean pain score was 3.7 ± 1.6, compared with 6.9 ± 1.7 in Group II (p < 0.05). Only six patients (30%) in Group I received opioids, and only one of these (5%) had a pain score >5. Conversely, 18 patients (90%) in Group II received Morphine during the first postoperative day.^11^ In our practice, Morphine has not been used for pain relief, as Dipyrone or other analgesics have been deemed sufficient.

In a randomized single-blinded study, 39 consecutive patients scheduled for thyroid surgery were assigned into two groups: Group I (n = 19) received preincisional subcutaneous infiltration with 10 mL of Bupivacaine 0.5%, and Group II (n = 20) received no infiltration. Postoperatively, pain was evaluated by verbal response scores and linear analogue scores (0–100 mm) at different time intervals following surgery. Although pain scores were significantly different at 6 h postoperatively (p = 0.0341), with mean scores of 33 and 50 in groups I and II, respectively, this difference dissipated at 24 h. None of the patients in Group I received IV Morphine, compared to 5 patients (25%) in Group II.^12^ We also observed a significant decrease in pain at the 24th postoperative hour compared to the two previous evaluation periods. Therefore, there is consensus that the pain relief is more pronounced in the early postoperative timeframe.

Another randomized, double-blind study assessed whether wound infiltration with Bupivacaine reduces postoperative pain and the need for analgesics after thyroid surgery. The patients were randomly divided into two groups and underwent the same analgesia protocol. An independent anesthesiologist prepared an unmarked syringe with either 10 mL of saline or 10 mL of 0.5% Bupivacaine. Just after the operation, patients were asked a blinded investigator to rate pain on a 0–100 mm VAS every 10 min. When the VAS score was ≥40 mm, a bolus of 2 mg of Morphine was injected, with reassessment every 10 min until a VAS score <40 mm was achieved. Pain scores were significantly lower in the Bupivacaine group at the 1 st postoperative hour and at 6, 12, and 24 h postoperatively.^8^ We chose not to inject saline into our control group. Nonetheless, our findings on pain outcomes were similar.

In contrast, another prospective randomized, double-blind study evaluated three groups: Group A – with Bupivacaine, Group B – with Bupivacaine with adrenaline, and Group C – control group. The average VAS scores in both study groups were comparable, and the difference was not statistically significant, indicating that wound infiltration with 0.5% Bupivacaine, with or without adrenaline, does not decrease pain after thyroidectomy.^5^

The referred pain after thyroidectomy originates from the cervicotomy, intra- operative neck hyper-extension that causes cervicalgia, as well as larynx irritation and discomfort resulting from tracheal stimulation caused by movement of the endotracheal tube during surgical manipulation. Additionally, pain can also be caused by the cervical drain, which usually remains in the OW for 24 h.^13^

In our study, as time progressed, it was observed that in the group that underwent infiltration, there was gradual pain relief, with the difference maintained for 24 h.

## Conclusion

Postoperative pain in all periods of observation was greater in the group that did not receive the anesthetic infiltration with Bupivacaine, underscoring the importance of considering topical anesthetic infiltration of the surgical scar as a tool against post-thyroidectomy pain.

## Declaration of competing interest

The authors declare no conflicts of interest.
